# Identification of Non-Canonical Translation Products in *C. elegans* Using Tandem Mass Spectrometry

**DOI:** 10.3389/fgene.2021.728900

**Published:** 2021-10-25

**Authors:** Bhavesh S. Parmar, Marlies K. R. Peeters, Kurt Boonen, Ellie C. Clark, Geert Baggerman, Gerben Menschaert, Liesbet Temmerman

**Affiliations:** ^1^ Animal Physiology and Neurobiology, University of Leuven (KU Leuven), Leuven, Belgium; ^2^ Laboratory of Bioinformatics and Computational Genomics (BioBix), Department of Mathematical Modelling, Ghent University, Ghent, Belgium; ^3^ Centre for Proteomics (CFP), University of Antwerp, Antwerp, Belgium

**Keywords:** *C. elegans*, altORFs, LC-MS/MS, mass spectrometry, timsTOF, MSFragger, PEAKS, sORFs

## Abstract

Transcriptome and ribosome sequencing have revealed the existence of many non-canonical transcripts, mainly containing splice variants, ncRNA, sORFs and altORFs. However, identification and characterization of products that may be translated out of these remains a challenge. Addressing this, we here report on 552 non-canonical proteins and splice variants in the model organism *C. elegans* using tandem mass spectrometry*.* Aided by sequencing-based prediction, we generated a custom proteome database tailored to search for non-canonical translation products of *C. elegans*. Using this database, we mined available mass spectrometric resources of *C. elegans*, from which 51 novel, non-canonical proteins could be identified*.* Furthermore, we utilized diverse proteomic and peptidomic strategies to detect 40 novel non-canonical proteins in *C. elegans* by LC-TIMS-MS/MS, of which 6 were common with our meta-analysis of existing resources. Together, this permits us to provide a resource with detailed annotation of 467 splice variants and 85 novel proteins mapped onto UTRs, non-coding regions and alternative open reading frames of the *C. elegans* genome.

## Introduction

Translation is a key biochemical process that produces a functional protein out of an open reading frame (ORF). While alternative definitions of an ORF exist (Sieber et al., 2018; Fermin et al., 2006; Claverie, 1997), we here use the term to indicate any mature mRNA sequence contained between a START and a STOP codon. With the advent of high-throughput sequencing and advanced computation, an *ad hoc* rule was established to restrict genomic annotation of ORFs to >100 codons unless previously characterised, as the small ORFs <100 codons (sORFs) posed higher probability of being false positives or biologically meaningless (Basrai et al., 1997). Moreover, most classical genome annotation pipelines enforce a stringent rule for monocistronic annotation of the longest possible ORF within an mRNA, further omitting sORFs and alternative ORFs (altORFs) beyond codon length restriction (Brunet et al., 2018). However, these notions have since been challenged with increasing evidence of non-canonical translation across eukaryotic life (Crowe et al., 2006; Kastenmayer et al., 2006; Ladoukakis et al., 2011).

For assignment of sORFs and altORFS, several bioinformatic and machine learning tools have been developed to predict 3- and 6- frame *in-silico* translation (Omasits et al., 2017; Brunet et al., 2018; Guruceaga et al., 2020). In addition, advances in ribosome sequencing have aided accurate annotation of codon triplet periodicity and non-AUG start sites, increasing the potential translatome (Mackowiak et al., 2015; Hao et al., 2018; Cesnik et al., 2020). Consequently, ORF annotation has gradually moved from the classical pipeline towards a larger theoretical premise that includes prediction of non-canonical translations from high resolution nucleotide sequencing and efficient signal scoring algorithms. Such ORF annotation pipelines provide searchable databases for discovery proteomics, of which databases following the classical pipeline tend to be more concise than non-canonical sORFs and altORFs prediction databases.

One of the principal tools in proteomics is mass spectrometry (MS), which works in conjunction with proteome databases. In bottom-up proteomics, the mass spectra of (often tryptic) peptides are matched against their *in silico* digested counterparts generated from a database. Under a broader proteogenomic framework, various computational strategies have been developed to integrate proteomic data with (canonical and non-canonical) genomic annotation pipelines or to generate standalone *in silico* translation databases for discovery of novel proteins (Risk et al., 2013; Jagtap et al., 2014; Mackowiak et al., 2015; Nagaraj et al., 2015; Zickmann and Renard, 2015; Kolmogorov et al., 2016; Olexiouk et al., 2016; Brunet et al., 2018; Guillot et al., 2019). At the MS-based experimental front, various fractionation and small protein enrichment methods have been employed to successfully identify novel non-canonical proteins in eukaryotic cell lines and tissues (Ma et al., 2016a; Li et al., 2017; He et al., 2018; Cao et al., 2020; Cardon et al., 2020; Kaulich et al., 2020; Cassidy et al., 2021; Wang et al., 2021). Together, the advances in genome annotation pipelines and high throughput mass spectrometry highlight the importance of both computational and technical approaches to successfully identify novel proteins. With massive data generated from nucleotide sequencing and mass spectrometry, refined annotation of model species genomes has been an ongoing endeavour in upgrading our understanding of molecular processes and discovering missing pieces.

*C. elegans* is an invertebrate model organism widely used for fundamental biological research (Brenner, 1974). In classical terms it has a well-annotated genome, yet little is known about its non-canonical content (Mackowiak et al., 2015; Casimiro-Soriguer et al., 2020). With more than 1,500 publications annually for the past decade, *C. elegans* research nonetheless benefits from extensive resources of sequencing and mass spectrometry data. The current Ensembl (release 101-August 2020) *C. elegans* annotation comprises approximately 61,000 transcripts classified as protein coding (20,000), non-coding (25,000), or - nonetheless - arising from presumed pseudogenes (2,000) (Yates et al., 2020). Two evidence-mapping pipelines utilizing sequencing-based predictions and *C. elegans* LC-MS/MS datasets identified 9 novel sORFs (Mackowiak et al., 2015) and about a 100 alternative ORFs (Brunet et al., 2018), respectively. However, no dedicated proteomic investigation has focused on profiling the non-canonical proteome in *C. elegans*. To our knowledge, the only focused research of sORFs in *C. elegans* originates from an evolutionary conservation-based genetic screen of intergenic ORFs wherein the authors identified 82 novel proteins (Casimiro-Soriguer et al., 2020).

We here report on the identification of 552 novel proteins within the *C. elegans* proteome. Combining a meta-analysis of available data with mass spectrometry-based discovery of protein extracts prepared especially for this purpose, we present a repertoire of novel *C. elegans* proteome members, including several with orthologs in other model organisms. These results provide a valuable resource for functional biological research using model organisms.

## Materials and Methods

### Construction of allORF Database

To identify unannotated novel proteins in the *C. elegans* proteome, an allORF database was constructed by complementing this organism’s reference proteome (Ensembl version 97, downloaded on August 21, 2019) with its alternative proteome predicted by two publicly available repositories, sORFs.org (Olexiouk et al., 2016; Olexiouk et al., 2018a) and Openprot (Brunet et al., 2019).

To obtain the putative coding sequences according to the method in sORFs.org, the public ribo-seq datasets (GSE62859 (Arnold et al., 2014), GSE52910 (Hendriks et al., 2014), GSE67387 (Nedialkova and Leidel, 2015), GSE65948 (Aeschimann et al., 2015)) were downloaded from NCBI Gene Expression Omnibus or NCBI Sequence Read Archive (SRA055804 (Stadler and Fire, 2011), SRA049309 (Stadler et al., 2012)) and processed according to a previously described pipeline (Olexiouk et al., 2018a) with minor adaptations. Here, raw reads were aligned using the STAR splice site aware mapper on the reference genome retrieved from the iGenomes repository with a P-site pinpointed by Plastid (Dunn and Weissman, 2016). After quality assessment with the mQC tool (Verbruggen and Menschaert, 2019), translation initiation sites were delineated using only the data of elongating ribosomes (Olexiouk et al., 2018a). Subsequently, sORFs with a maximum length threshold of 100 codons were assembled by the previously published sORFs.org pipeline with minor code modifications (Olexiouk et al., 2018b). To take the compact genome of *C. elegans* into account, the noise filtering settings were set at alpha = 0.2. Only the longest sORF of candidates which shared a stop site was retained in order to reduce redundancy. Finally, duplicated sequences were removed to obtain the final database of predicted putative sequences based on the sORFs.org method. To reduce the sORF predictions overlapping with known or predicted proteins longer than 100 amino acids, the sequences of the predicted sORFs (excluding the first and the last amino acid) were tested for identical overlap with the protein sequences of the reference Ensembl and the *C. elegans* AltProts and Isoforms from the Openprot repository (Brunet et al., 2019) (release 1.3, downloaded 5th of November 2019) with an in-house scripting module (written in *Python*, available upon request). Finally, the reference Ensembl, the downloaded OpenProt, and the filtered sORFs.org predictions were concatenated with the cRAP database (downloaded on 6th of November 2019) containing commonly identified contaminants in MS analysis, and the proteome of *E. coli* (strain K12, version 45, downloaded on 6th of November 2019) to account for contaminants introduced by the feeding conditions to obtain the final allORF search database.

### Evaluation of allORF Database Characteristics

The features of the proteins present in the different database parts of the final allORF database were analysed using the Peptides package (Osorio et al., 2015) in R 4.0.2 (R Core Team, 2020) and visualized with the ggplot2 package (Wickham, 2016) (Supplementary Datasheet 5 Figure S1).

The final allORF database was *in silico* digested by trypsin allowing one missed cleavage and a mass limit between 600 and 4,000 Da, followed by redundancy clearance using the DBToolkit 2.0 (Martens et al., 2005) (version 4.2.5, downloaded on the August 11, 2020). Subsequently, only non-redundant peptides with a length between 7 and 30 amino acids were kept and mapped against the allORF database to identify the number of unique *in silico* peptides per alllORF protein. Finally, the results were visualized with the ggplot2 package (Wickham, 2016).

### Worm Culture

All animals (*C. elegans* LSC 1918) were cultured at 20 °C on Nematode Growth Medium plates supplemented with *E. coli* OP50 as described previously (Lewis and Fleming, 1995).

### Construction of HiBit Strain LSC1918

A nucleotide sequence coding for the 11 amino acid HiBit tag (gtg​agc​ggc​tgg​cgc​ctg​ttt​aaa​aaa​att​agc) was inserted at the C-terminus of an 84 amino acid annotated protein R06C1.4.1 (strain: LSC 1917) and a predicted 59 amino acid altORF on C05C9.3 (Openprot ID: IP_1,500,296, strain: LSC 1916), using the CRISPR-cas9 system with the *dpy-10* co-CRISPR marker as described previously (Paix et al., 2017); all oligo sequences can be found in Supplementary Datasheet 5 Table S1. Briefly, guide RNA was designed based on sequence scoring (Integrated DNA Technologies CRISPR design checker tool) and proximity to the R06C1.4.1 and IP_1,500,296 stop codon. The repair template comprised ∼35bp DNA homology arms with the HiBit nucleotide sequence inserted before the stop codon. The injection mix comprised 2.5 µL Cas9 enzyme (15 μg/μL), 2.5 µL tracrRNA (0.17 nmol/μL), 1 µL *dpy-10* crRNA (0.6 nmol/μL), 1 µL R06C1.4.1 or IP_1,500,296 crRNA (0.6 nmol/μL), 1 µL *dpy-10* repair template (0.5 μg/μL), 1 µL R06C1.4.1 or IP_1,500,296 repair template (1 μg/μL). For LSC 1916, injected hermaphrodites were allowed to lay eggs, F1 offspring were singled out based on the roller phenotype characteristic of heterozygous insertion of the *dpy-10* co-CRISPR marker and cultured until sufficient F2 progeny were present. For LSC 1917, injected hermaphrodites were allowed to lay eggs, F1 offspring were singled out based on the dumpy phenotype characteristic of homozygous insertion of the *dpy-10* co-CRISPR marker and cultured until sufficient F2 progeny were present. Each singled out F1 worm from LSC1916 and LSC1917 was then lysed, and PCR verified for HiBit sequence insertion using digestion of the PCR product with Mbil (Thermo fisher, FastDigest) as per the manufacturer’s protocol. The HiBit nucleotide sequence contains the MbiI target cleavage sequence, hence, successful digestion is a readout for successful integration of the HiBit repair template. LSC1916 non-roller progeny (F2) of the HiBit-containing roller F1 were singled out to restore the co-CRISPR locus to its wild type allele, cultured, and screened for homologous HiBit insertion using PCR amplification and enzymatic digestion as described above. LSC1916 HiBit insertion strain with wild-type background was then crossed (Fay, 2006) with homozygous LSC1917 HiBit insert with dumpy background and progeny were screened for homologous HiBit insertion on both locus with restored wild-type co-CRISPR locus. The resulting LSC1918 strain was verified by sequencing the genomic region of R06C1.4.1 and C05C9.3 containing the Hibit sequence (Supplementary Datasheet 5 Table S1).

### Worm Sampling and Protein Extraction

LSC1918 worms were synchronized by standard hypochlorite treatment (Porta-De-La-Riva et al., 2012). Following overnight incubation in S-basal (5.85 g NaCl, 1 g K_2_ HPO_4_, 6 g KH_2_PO_4_ in 1 L milliQ), L1 arrested animals were cultured on Nematode Growth Medium plates (Nematode Growth Medium (NGM), 2014) with an *E. coli* OP50 lawn (∼3,000 worms/plate, 5 plates/sample), at 20°C for 52 h, which is until the worms had reached the young adult stage. Worms were washed off the plates with S-basal and allowed to settle in 15 ml tubes for 10 min at room temperature. To remove bacteria, the pellet was washed three times with S-basal while allowing the worms to settle for 5 min after each wash. After a final wash with ultrapure (Milli-Q) water, worm pellets were snap frozen in liquid nitrogen and stored at −80°C until further use.

For protein extraction, the frozen pellet was thawed by adding a double volume of lysis buffer (8 M urea, 2 M thiourea, 1 mM dithiothreitol, 1x cOmplete™ protease inhibitor cocktail (Roche)). The mixture was first homogenised in a Precellys-Cryolys homogenizer (Bertin Instruments) using an equal volume of ceramic beads (1.4 mm zirconium oxide, Bertin Technologies) beaten at 6,800 rpm for 10 cycles of 10 s each, with 20 s pause between each cycle at a temperature below 4°C. The homogenised lysate was collected and further sonicated on ice, using a probe sonicator for 12 cycles (5 s ON, 10 s OFF). The lysate was then spun at 16,000 *g* for 30 min at 8°C. The supernatant was collected and protein concentration of samples was estimated using a standard Bradford assay (Harlow and Lane, 2006).

### Enrichment of Low Molecular Weight Proteins

Broadly, two strategies were employed for enrichment of low-molecular weight proteins and peptides, *viz.* (C8 and C18) reversed phase chromatography and gel electrophoresis using Tris-Tricine SDS-PAGE. Enriched fractions were either enzymatically digested (with trypsin or chymotrypsin) or loaded undigested onto the mass spectrometer after C18 cleanup. Additionally, 20 µg worm lysate pre-enrichment was also digested for assessment of enrichment strategies compared to a whole-sample shotgun proteomic approach. All experiments were conducted with four biological replicates.

A) **Whole mount digestion**: 20 µg worm lysate pre-enrichment was reduced with 5 mM dithiothreitol at 56°C for 30 min, alkylated with 25 mM iodoacetamide at room temperature in the dark for 20 min. The volume was adjusted to 1 M urea with 50 mM triethyl ammonium bicarbonate and digested overnight with 1 µg of trypsin (at 37°C) or chymotrpysin (at RT) (Promega BNL, Netherlands). The reaction was then stopped by acidifying the samples to 0.1% formic acid. Following that, the sample was cleaned using C18 spin columns (Pierce™). Briefly, the column was rinsed with 50% methanol, equilibrated 3 times with 200 µL of 5% acetonitrile, 0.1% formic acid by spinning at 1,500 *g* for 1 min. The digested sample was loaded, washed 4 times with 5% acetonitrile, 0.1% formic acid and eluted in 50 µL of 30% acetonitrile and 50 µL of 60% acetonitrile with 0.1% formic acid, and dried using a Savant SpeedVac concentrator.
B) **C8 reversed phase enrichment**: Bond Elut C8 solid phase extraction cartridges (Agilent Technologies, United States) were coupled with a vacuum manifold with the pressure set to 1,000 mbar. The column was first rinsed with 6 ml of 50% methanol and equilibrated thrice with 6 ml buffer A (20 mM ammonium acetate in Milli-Q, pH 7.0). 2.5 mg of protein lysate was diluted 1:7 with buffer A and samples were loaded onto the equilibrated sorbent at room temperature and left undisturbed for 4 min. Following that, the sample was allowed to flow through the sorbent and the column was washed five times with 6 ml buffer A. Bound proteins and peptides were eluted with 3 ml of buffer B (75% acetonitrile in 20 mM ammonium acetate pH 7.0). The eluent was dried in a SpeedVac concentrator (Savant) and redissolved in 100 µL of 50 mM triethyl ammonium bicarbonate. Recovery concentration was estimated using a Bradford assay (Harlow and Lane, 2006). 10 µg of sample were reduced with 5 mM dithiothreitol at 56°C for 30 min and alkylated with 25 mM iodoacetamide at room temperature in the dark for 20 min. Samples were then digested overnight with 0.5 µg of trpysin (at 37°C) or chymotrpysin (at room temperature) (Promega, Netherlands). The reaction was stopped by acidifying the samples to 0.1% formic acid. 20 µg of undigested C8-enriched sample were also acidified to 0.1% formic acid and cleaned to capture peptides not amenable to bottom-up proteomics. The cleanup for digested and undigested peptides was performed as described above (*cf.* A).
C) **Acid precipitation enrichment:** for enrichment of peptides on C18, we adapted the protocol previously published by Secher et al. (2016). 500 µg of lysate were acidified to 0.5% acetic acid, vortexed for 1 h at 4°C and spun at 16,000 *g* for 15 min at 4°C. The supernatant was collected and filtered through a 10,000 Da (Da) molecular weight cut-off filter for 20 min at 4,000 *g* at 4°C (Amicon^®^ Ultra-4 centrifuge filters, Merck Millipore, pre-rinsed twice with 50% methanol). The flow-through was then cleaned on C18 spin columns as described above (*cf.* A).
D) **Tris-tricine SDS PAGE and in-gel digestion**: 200 µg of lysate was run on 16.5% tris-tricine SDS-PAGE (Criterion™, Biorad) at 150 V for 30 min. The gel was washed twice with Milli-Q and the unstained gel was cut using a sterile scalpel to collect the fraction between 2,000 and 12,000 Da according to the Precision Plus Protein™ Dual Xtra Prestained Protein Standards (Bio-Rad). The gel fraction was diced into 1 mm^3^ pieces, washed with 500 µL 50% acetonitrile in 25 mM ammonium bicarbonate and dehydrated with 100% acetonitrile by vortexing for 10 min. The dehydrated gel pieces containing proteins were allowed to rehydrate in 25 mM ammonium bicarbonate with 5 mM dithiothreitol and incubated at 56°C for 30 min. Unabsorbed buffer was removed and replaced with 25 mM iodoacetamide in 25 mM ammonium bicarbonate and incubated at room temperature in the dark for 45 min. Following that, the gel pieces were dehydrated again with 100% acetonitrile and rehydrated for 30 min on ice with 200 µL 25 mM ammonium bicarbonate, 10% acetonitrile and 3 µg of trypsin or chymotrypsin (Promega, Netherlands). 25 µL of 25 mM ammonium bicarbonate were added to cover the gel pieces, which were then incubated overnight at 37°C (trypsin) or room temperature (chymotrypsin). The following day, the unabsorbed mixture was collected in a fresh LoBind tube (Eppendorf), and digested peptide was extracted from the gel pieces by vortexing in 300 µL of 80% acetonitrile, 5% formic acid for 30 min. The extract was pooled with the unabsorbed mixture and the sample was dried using a Savant SpeedVac concentrator. The dried peptides were redissolved in 5% acetonitrile, 0.1% formic acid and cleaned as described above (*cf.* A).

### LC-MS/MS

The sample was dissolved in 10 µL of 6% ACN and 0.1% FA and separated on a ACQUITY UPLC M-Class System (Waters), fitted with a nanoEase™ M/Z Symmetry C18 trap column (100 Å, 5 μm, 180 μm × 20 mm) and a nanoEase™ M/Z HSS C18 T3 Column (100 Å, 1.8 µm, 75 μm × 250 mm, both from Waters). The sample was loaded onto the trap column in 2 min at 5 μL/min in 94% buffer A, 6% buffer B (buffer A is 0.1% FA in MilliQ, buffer B 0.1% FA in 80% ACN). The flow over the main column was 0.4 μL/min and the column was heated to 40°C. After an isocratic flow of 4 min at 6% B, the concentration of B increased in 36 min–50% B, then to 94% B in 4 min, using linear gradients. After again an isocratic flow of 4 min at 94% B, the concentration of B decreased in 4 min–6% which was followed by 15 min of equilibration at an isocratic flow of 6% B. The column was online with a timsTOF Pro operating in positive ion mode, coupled with a CaptiveSpray ion source (both from Bruker Daltonik GmbH, Bremen). The timsTOF Pro was calibrated according to the manufacturer’s guidelines. The temperature of the ion transfer capillary was 180°C. The Parallel Accumulation–Serial Fragmentation DDA method was used to select precursor ions for fragmentation with 1 TIMS-MS scan and 10 PASEF MS/MS scans, as described by Meier et al. (2018). The TIMS-MS survey scan was acquired between 0.70–1.45 V s/cm^2^ and 100–1700 m/z with a ramp time of 166 ms. The 10 PASEF scans contained maximum of 12 MS/MS scans per PASEF scan with a collision energy of 10 eV. Precursors with 1 – 5 charges were selected with the target value set to 20,000 a. u. and intensity threshold to 2,500 a. u. Precursors were dynamically excluded for 0.4 s. The timsTOF Pro was controlled by the OtofControl 5.1 software (Bruker Daltonik GmbH). 10 PASEF scans contained on average 12 MS/MS scans per PASEF scan. Raw data were analysed with the DataAnalysis 5.1 software (Bruker Daltonik). All mass spectrometry raw and spectrum files can be downloaded from MassIVE with identifier MSV000087909.

### Data Analysis

For meta-analysis of proteomic datasets of larval, adult and Stress *C. elegans*, raw files were accessed from ProteomeXchange (http://www.proteomexchange.org/) via their respective IDs (PXD006676 (Xia et al., 2018), PXD004584 (Narayan et al., 2016), PXD005649 (Edifizi et al., 2017)). The raw files were then analysed on PEAKS Studio X (Bioinformatics Solutions Inc., Canada) with precursor tolerance set to 10 ppm and a fragment tolerance of 0.02 Da with fully tryptic digestion and 2 allowed missed cleavages. The fixed and variable modifications were set according to the information published in the respective original studies. For experiments conducted in this study using LC-TIMS-MS/MS, data were analysed using MSFragger based Fragpipe (Yu et al., 2020a) and PEAKS Online Xpro (Bioinformatics Solutions Inc., Canada). For C8 enriched, in-gel digested and whole-sample digests, carbamidomethylation (C, +57.02) was set as a fixed modification and carbamidomethylation (DHEK &N-term, +57.02), N-terminal acetylation (+42.01), oxidation (M, +15.99), pyroglutamation (N-term E, -18.01), pyroglutamation (N-term Q, -17.02) and deamidation (NQ, +0.98) were set as variable modifications with only 3 allowed modifications per peptide. The enzyme (trypsin or chymotrypsin) was chosen corresponding to the respective digests, with full specificity. Precursor tolerance was set to 20 ppm and fragment tolerance was set to 0.05 Da. For undigested samples, variable modifications were N-terminal acetylation (+42.01), oxidation (M, +15.99), pyroglutamation (N-term E, -18.01), pyroglutamation (N-term Q, -17.02) and deamidation (NQ, +0.98) with non-specific cleavage and no fixed modification. The d raw files were searched against our *C. elegans* allORF database (Supplementary Data Sheet 1.FASTA) on both the search engines and filtered to remove contaminants and non-razor (subgroup) proteins and only top group proteins were considered. Protein identifications with at least 1 peptide at 1% FDR were considered and further analysis was performed in R studio (http://www.rstudio.com/) using custom script for statistics and visualization. For Figure 3A,B, the data was fitted into a generalized linear mixed model and significance calculated with Type II Anova. Pairwise posthoc comparison was performed with least square means and Benjamini-Hochberg correction. For interaction statistics for non-canonical identifications (Figure 4B,D and Supplementary Datasheet 5 Figures S2B,D), superexact test (Wang et al., 2015) was performed. Other statistical tests conducted in this study are mentioned in the respectively text and/or caption along with resulting *p*-values.

### Custom BLASTp Candidate List

A local BLAST search database was created with the BLAST + application (Camacho et al., 2009) using the alternative proteome of four model organisms, namely fruit fly, human, house mouse and zebrafish, downloaded from Openprot (release 1.3, downloaded on 6th of September 2018), sORFs.org (downloaded on 6th of September 2018) and described by Mackowiak et al. (2015). The BLASTp algorithm was applied to search the candidate list against the individual search database of each model organism. For every search, only the hit with the lowest Expect value (minimum E = 10^−10^) and highest sequence identity per model and database was retained by manual inspection (Supplementary Datasheet 3).

## Results

### Construction of a *C. elegans* allORF Database

**FIGURE 1:**
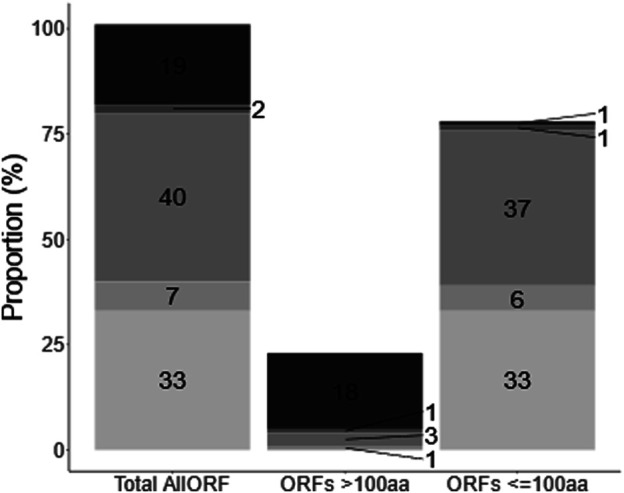
Composition of allORF database. The allORF database combines sequence information from Ensembl, Openprot and sORFs.org. (From top to bottom: Ensembl (19), Ensembl + sORFs.org (2), OpenProt (40), OpenProt + sORFs.org (7), sORFs.org (33). OpenProt + Ensembl + sORFs.org (3 ORFs on a total of 137,166 ORFs) and OpenProt + Ensembl (25 ORFs on a total of 137,166 ORFs) are not shown due to the low number). Numbers indicate the percentage of ORFs in the allORF database retrieved from each sequence source.) Second and third bar show the same information grouped proteins (>100 codons) and small (<100 codons) ORF types.

In this work, we set out to expand and describe the *C. elegans* non-canonical proteome. We built a custom database to provide a comprehensive proteomic search space, encompassing all theoretically possible translational outcomes based on available transcript and ribosome sequencing data. To that end, we used predictions from Openprot and sORFs.org concatenated with the Ensembl *C. elegans* proteome. Openprot predictions comprise altORFs and isoforms without codon length cut-off, whereas sORFs.org contributed to prediction of sORFs <100 codons. The resulting allORF database in total comprises 137,194 protein sequences (Figure 1 and Supplementary Datasheet 5 Table S2), which for this species is about five times more than the Ensembl data alone (25,886 protein sequences). Briefly, about 77% of the allORF database comprises small (<100 codons) ORF predictions from Openprot and sORFs.org, whereas 2% of the database are sORFs that originate from Ensembl. Additionally, about 18% of all ORFs are >100 codons and retrieved from Ensembl, with the remaining 4% also longer than 100 codons but derived from Openprot and sORFs.org (Figure 3 and Supplementary Datasheet 5 Table S2). For allORF database assembly, the calculated median ORF length within the sORFs.org predictions was 25 codons whereas for Openprot predictions, this was 45 codons (Supplementary Datasheet 5 Figure S1). With about 70% of allORF sequences unique to either one of these two prediction algorithms, the newly assembled database efficiently exploits the complementarity of Ensembl, sORFs.org and Openprot.

### The allORF database unveils 385 novel non-canonical proteins in published proteomics data of *C. elegans*

Despite stringent concatenation of redundancy in the allORF database, we anticipated that the sheer number of entries might increase false positives in proteomic mapping. To test this, we utilized available *C. elegans* raw data from Xia et al. (2018) (larval - PXD006676), Narayan et al. (2016) (adult - PXD004584) and Edifizi et al. (2017) (stressed - PXD005649) and re-analysed over 120 raw files of 240 min gradient runs each against our allORF database using PEAKS. These datasets are interesting from a discovery perspective because they cover a range of physiological changes within the worm: the larval dataset contains samples of all 4 larval stages (L1-L4), the adult dataset includes day 1, day 5 and day 10 adult worms, and stressed samples were subjected to UV-irradiation or starvation. In total, 10,651 proteins were identified across all three datasets with at least one unique peptide at 1% FDR (Supplementary Datasheet 1). This is in the same range as originally identified in the respective studies, with minor differences due to differences in database, analysis pipeline and because for the adult dataset, we only considered one biological replicate (with 80 LC-MS/MS runs).

**FIGURE 2:**
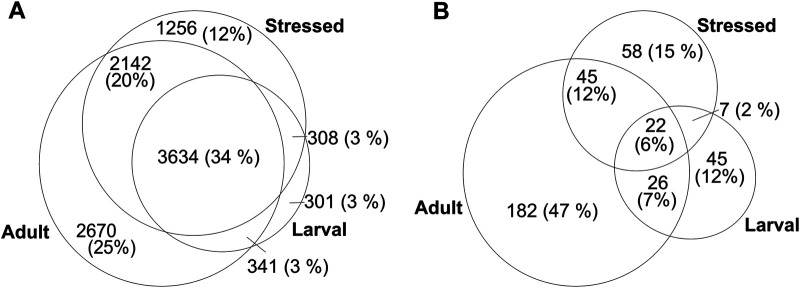
Numbers of identified proteins (grouped) in larval Xia et al., 2018, adult Narayan et al., 2016 and stressed Edifizi et al., 2017 *C. elegans* LC-MS/MS datasets, with at least 1 unique peptide at 1% FDR. **(A)** Total number of unique identifications **(B)** non-canonical proteins as predicted by Openprot and sORFs.org.

About 82.5% of the proteins were identified in the adult-stage dataset (Figure 2A). Although there is a clear complementarity of the diverse *C. elegans* sampling conditions that benefits discovery of novel proteins, this observation is likely due to technical differences. The adult dataset comprises five extraction methods and 80 fractions as compared to a shotgun approach used for the larval and Stress datasets, increasing the depth of identification in the adult *C. elegans* dataset. Of the 10,651 proteins in total, 385 identifications relied on either Openprot or sORFs.org contributions to the allORF database (Figure 2B). 85% of these non-canonical identifications categorize as non-canonical isoforms, whereas the remaining 51 novel proteins originate from genomic regions such as UTRs, ncRNA and polycistronic mRNA with alternative open reading frames (Supplementary Datasheet 3).

The experimental data used here were retrieved from studies that followed well-established bottom-up proteomic sample preparation strategies with depth achieved mainly by peptide fractionation (adult dataset) and long liquid chromatographic separation coupled online with orbitrap-based mass spectrometric detection. Thus, this meta-analysis of available data provided an efficient means for testing the efficacy of ORF predictions in our database, and of existing proteomic strategies to capture novel proteins.

### Enrichment Strategies and Alternative Cleavage for Shotgun Mass Spectrometry Analysis Are Complementary to Standard Proteomic Digest

Most non-canonical predictions reside in the low molecular weight range, with median length of 25 amino acids for sORFs.org and 45 amino acids for OpenProt predictions (Figure 1 and Supplementary Datasheet 5 Figure 1). To evaluate whether enrichment strategies could be beneficial for cost and time-efficient shotgun identification of novel non-canonical proteins, we combined eight different strategies with LC-TIMS-MS/MS (Supplementary Datasheet 5 Table S3). Next to using strategies described in literature, *viz.* Tris-tricine SDS-PAGE (Wang et al., 2021) and C8-reversed phase (Ma et al., 2016a) enrichments, we enzymatically digested the whole worm lysate under denaturing conditions. To further improve the protein sequence coverage and the expected depth of our analysis, we utilized complementary cleavage enzymes, trypsin and chymotrypsin, in combination with the three strategies (conditions named GelT, GelChT, C8T, C8ChT, WLT, WLChT henceforward). Finally, we also included undigested peptidome analysis of C8-reversed phase enriched samples (henceforward 2DRP) and an adaptation of the method by Secher et al. (2016), which aims at capturing endogenous peptides and peptides not amenable to enzymatic digestion due to their small size and lack of cleavage site (henceforward 1DRP).

**FIGURE 3:**
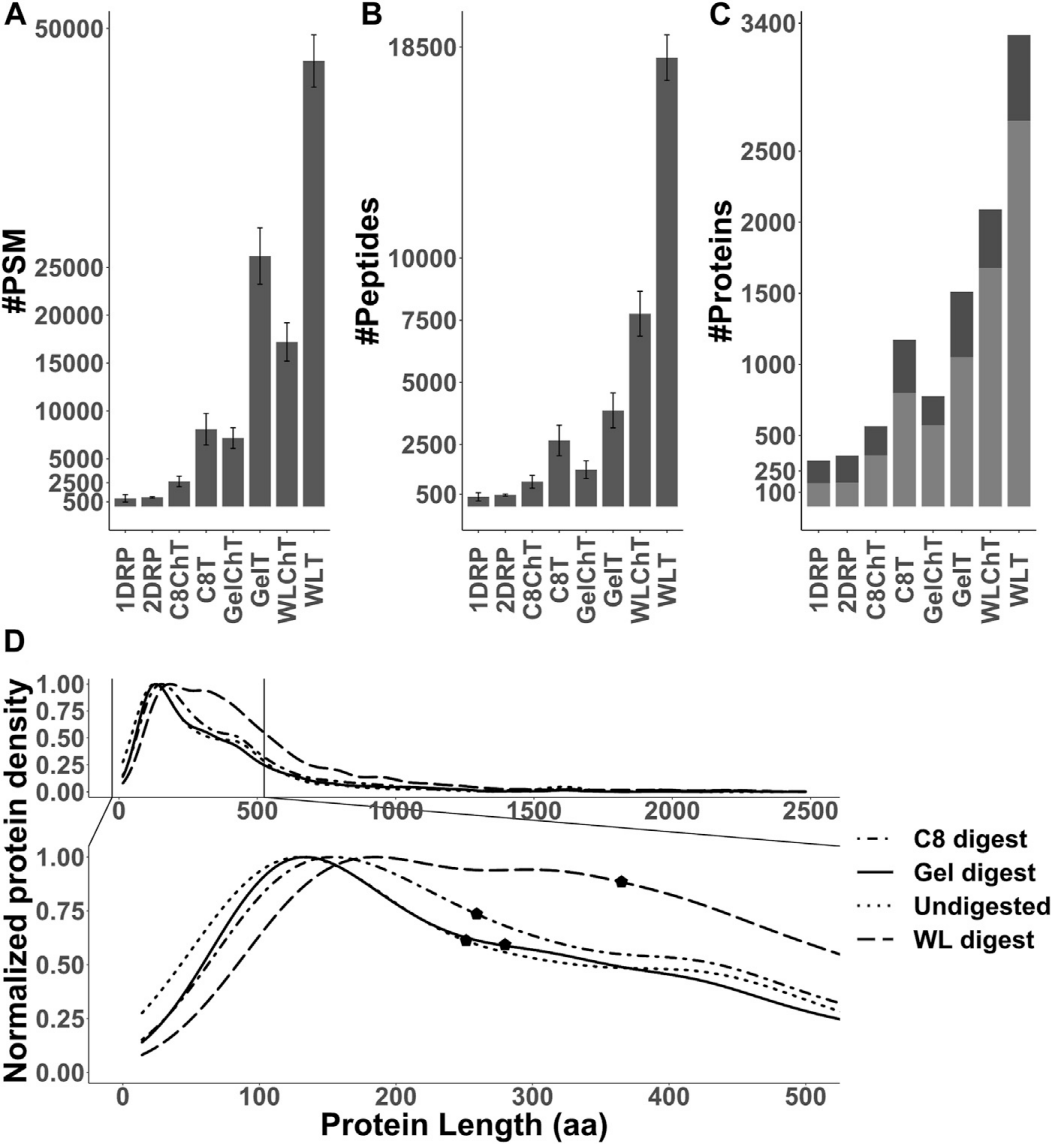
**(A,B)** Number of peptide spectrum matches (PSM) and peptides identified for each sample processing method used in this study (*p*-value <0.001). Error bars indicate standard deviation between replicates (*n* = 4). **(C)** Total number of proteins for all replicates combined, with unique (stripped) peptides >1 (light gray) or = 1 (dark gray) per condition. **(D)** Distribution of identified proteins and their length normalized to the total of identifications per group and median markers (p_Fligner-Killeen_: 2e-16).

**FIGURE 4:**
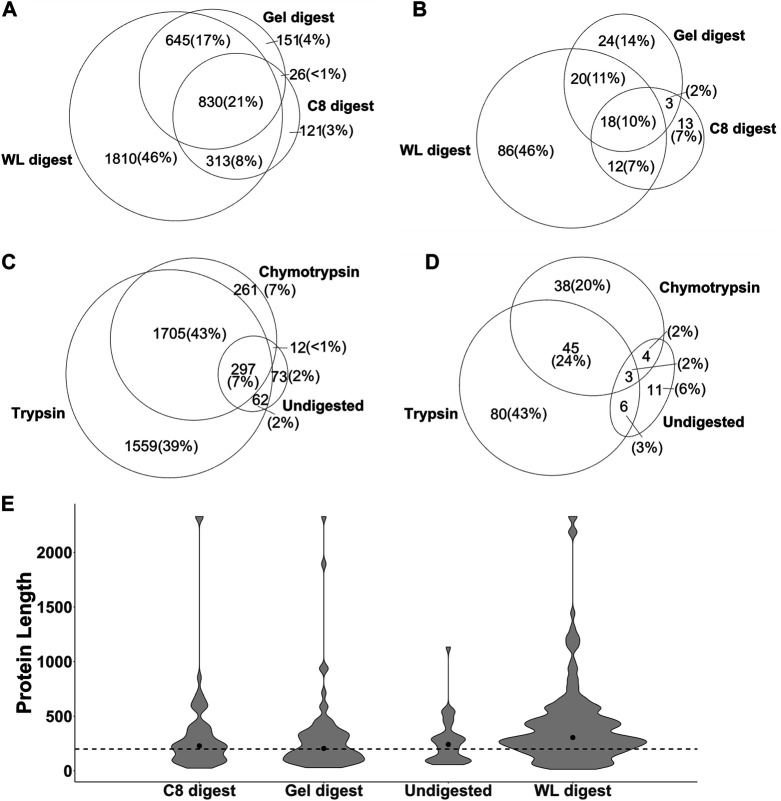
The identification of total proteins **(A,C)** and non-canonical proteins (**(B,D)** *p*-value: <0.001) is influenced by sample processing methods **(A,B)** and enzymatic cleavage **(C,D)**. WL: whole lysate, C8: C8-reversed phase enriched, Gel: in-gel digested samples, Undigested: 1DRP + 2DRP. **(F)** Absolute count of non-canonical proteins identified in each group and distribution along protein amino acids length with dotted line at 200 amino acids and median represented by a dot.

Out of the 8 strategies, the undigested fractions (1DRP, 2DRP) aimed at capturing endogenous peptides led to the least number of peptide spectrum matches (PSMs), peptides and protein identifications (Figures 3A–C). In the range of 2.5–12 kDa proteins, numbers from C8-enrichment and digestion with trypsin and chymotrypsin (C8T, C8ChT) were superseded by those of in-gel digestion (GelT, GelChT), indicating that a well-optimized in-gel digestion method is far more efficient at size separation of proteins compared to a standard C8 reversed-phase solid phase extraction (Figures 3A,B, all *p*-values < 0.001). As anticipated, the whole worm lysate digest (WLT, WLChT) yielded maximum identifications in total, whereas relatively lower numbers of proteins were matched from enriched samples (Figure 3C, 4A). Compared to whole lysate, the C8 digests, gel digests and undigested samples performed relatively better at targeting proteins below 150 amino acids (Figure 3D, *p*_*Fligner-Killeen*_: 2e-16). With WL, Gel, C8 and undigested runs combined, a total of 3,969 proteins groups were identified (Supplementary Datasheet 2). The WL digest (Tryptic + Chymotryptic) accounts for >90% of these identifications (Figure 4A) while only less than 10% were derived uniquely from enriched and digested samples. However, this ratio is increased more than 2-fold for non-canonical identifications, with 22.7% uniquely contributed by the total of all enrichment methods (Figure 4B). Enriched samples do contribute unique identifications, indicating that these methods may add distinctive complementarities to the WL digests. Our data also suggest that enzymatic digestion choices may influence peptide identification: undigested samples uniquely contributed to only 2% and chymotrypsin-digested to 6.8% of the total number of identifications, meaning less than 10% of the total went undetected in their tryptic counterparts (Figure 4C). However, chymotrypsin-treated and undigested peptidomes accounted for 53 unique identifications of the total 187 novel non-canonical proteins identified across all groups, underscoring the added value of experimental diversity in discovery approaches (Figure 4D). In absolute numbers, the novel non-canonical proteins identified across all conditions span a wide protein length range with median close to 200 amino acids for the total of all enriched samples (Figure 2E), with a significant distinction in non-canonical proteome coverage depending on the sample type (Supplementary Datasheet 5 Figure S2E, *p*_*Kruskal-Wallis*_ 0.01). Together, enrichment for low molecular weight proteins and an alternative cleavage strategy contributed to a quarter of all novel non-canonical proteins identified in our experiments. Moreover, trypsin - the workhorse enzyme for bottom-up proteomics - outperforms chymotrypsin in the number of peptides and protein identifications across sample processing conditions (Figure 3B,C), but each enzyme also contributes unique identifications of novel non-canonical peptides to our resource (Figure 4D). Enrichment strategies facilitated the identification of an additional 305 proteins (8.4% of total) for tryptic (Supplementary Datasheet 5 Figure S2A) and 185 proteins (8.1% of total) for chymotryptic (Supplementary Datasheet 5 Figure S2B) digests, that were missed in their whole lysate digested counterparts. The advantage of enrichment with SDS-PAGE and C8-reversed phase is more prominent for non-canonical identifications, with a distinct complementarity achieved in either case in comparison to whole lysate digests (Supplementary Datasheet 5 Figures S2C,D).

### Alternative Search Engines Further Improve the Detection of Novel Proteins

**FIGURE 5:**
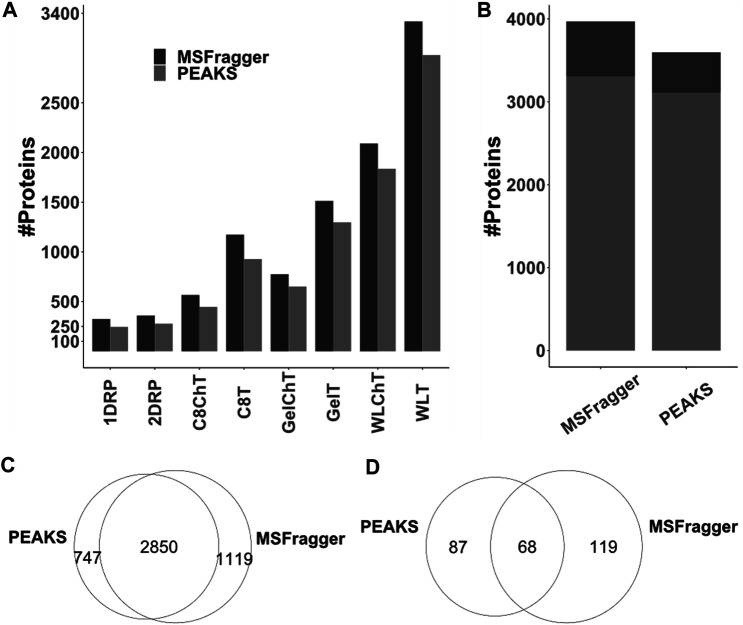
Comparison of PEAKS and MSFragger protein identifications for LC-TIMS-MS/MS data acquired in this study **(A)** total protein numbers as identified per condition **(B)** combined total protein numbers across all conditions for proteins with unique (stripped) peptides >1 (light gray) and >1 (dark gray). **(C,D)** While there is certainly overlap, each algorithm contributes unique identifications to the list of total **(C)** and non-canonical **(D)** proteins.

Multiple peptide search algorithms exist to aid efficient mapping of experimental LC-MS/MS data against proteome databases; however, underlying principles and data training may differ widely. For example, MSFragger (Kong et al., 2017) employs a fragment ion indexing method and supports open searches for mapping peptides onto a database, whereas PEAKS (Zhang et al., 2012) generates *de novo* tags from MS data for peptide identification and prediction of post-translational modifications. To test whether differences in algorithm would affect the identification success, hence discovery of novel peptides, we processed the same raw files (*i.e.,* 32 × 60 min runs) already analysed via MSFragger (*cf.* above) with PEAKS Online Xpro using the same parameters. Across all sample processing conditions, the number PSMs (Supplementary Datasheet 5 Figure S3A) and unique peptide sequences (Supplementary Datasheet 5 Figure S3B) identified by MSFragger and PEAKS differed slightly. However, MSFragger consistently identified more proteins across all sample processing conditions (Figure 5A, Supplementary Datasheet 5 Table S4). For combined results from all the experiments in this study, the difference in sheer total number of identified proteins between the two search engines is 372 (approximately 10%) with relatively more proteins identified based on only 1 unique peptide by MSFragger (Figure 5B, dark gray). A huge difference is observed in distinct protein identifications reported by the two search algorithms (Figure 5C) with 747 and 1,119 unique proteins with at least 1 peptide identified by PEAKS *vs* MSFragger, respectively. Similarly, 87 unique non-canonical proteins were added by PEAKS to our previous list of 187 from MSFragger, with 68 common between the two search algorithms, taking the total count of non-canonical proteins identified in this study to 274 (Figure 5D). As was the case for complementary sample processing, a complementarity is observed in data analysis pipelines as well. Although similar strategies have been utilized previously and search engines do perform differently (Shteynberg et al., 2013), the differences observed in our study amount to nearly 40% of the total protein identifications that are unique to either of the search engines. This suggests that efficient spectrum matching algorithms are yet to be standardized for search engine-wide consistency and the combination of search engines is likely to be more informative.

#### Annotation of Identified Novel Non-Canonical Proteins

**FIGURE 6:**
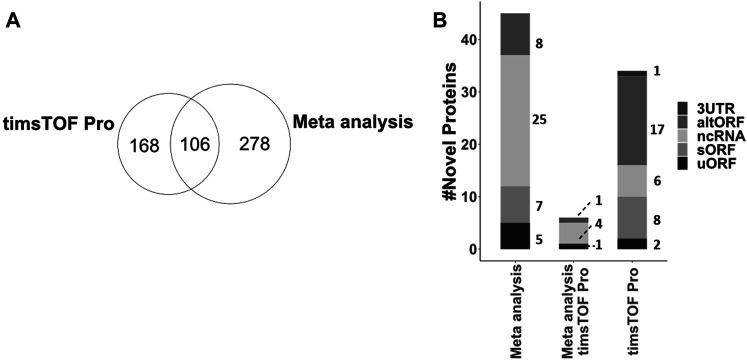
**(A)** Comparison of all 552 non-canonical proteins, including 467 splice variants and 85 novel proteins, identified from meta-analysis of larval, adult and Stress proteomic datasets acquired on Orbitrap platform and experiments conducted in this study on timsTOF Pro platform. **(B)** Annotation of 85 novel proteins identified either via meta-analysis, timsTOF Pro acquisition of our experiments or both, mapped on C. elegans genome.

Using a custom *C. elegans* database, existing *C. elegans* LC-MS/MS data and experiments aimed at capturing non-canonical proteins, we report the identification of 552 non-canonical proteins based on predictions from Openprot and sORFs.org. 324 out of these were identified with more than 1 unique peptide, 42 with 1 unique peptide but across multiple samples, and 186 with only 1 unique peptide. Of the total of non-canonical proteins identified in this study, 106 identifications were common between the samples analysed with timsTOF Pro and the meta-analysis performed on three proteomic datasets (acquired via Orbitrap platform), while 168 proteins were unique to our experiments and 278 mined from pre-existing *C. elegans* datasets via our meta-analysis (Figure 6A). 467 of these proteins correspond to low-novelty isoforms/splice variants. From the remaining 85, 8 were mapped onto 5′UTR (uORF) of annotated genes, 1 in 3′UTR, 35 in non-coding RNA (ncRNA), and 41 are alternative polycistronic translation products from annotated genes (15 sORF and 26 altORF respectively, Supplementary Datasheet 3). This group represents the novel proteins that are crucial for further investigation and functional annotation. To further assess the significance of this group from a model organism perspective, we searched these 85 candidates against the human and three other model organism proteomes (*H. sapiens*, *M. musculus*, *D. melanogaster* and *D. rerio*) via a custom BLASTp script. 18 out of 85 were found to be conserved across these species (Supplementary Datasheet 3). Together with the less-conserved novel proteins and protein variants, these non-canonical proteins add to the proteogenomic repertoire of interest for functional genetics research.

## Discussion

Protein translation is a major driver of organismal phenotypes and plasticity, and for decades now, breakthroughs in all domains of biological sciences have relied on understanding (differences in) translation - hence, indirectly on genome annotation. However, genome annotation is mired with inaccuracies and many genomes, as well as the process of annotation itself, are iteratively updated based on new evidence. In recent years, increased interests in non-canonical translation products such as sORF- and ncRNA-encoded peptides or proteins have motivated researchers to revisit the full complexity of functional genome annotation. Given the anomalous nature of their coding potential and small size, non-canonical ORFs tend to escape the annotation radar. This is also true for the otherwise well-annotated genome of the model organism *Caenorhabditis elegans*. In an effort towards unveiling non-canonical translation products in *C. elegans*, we therefore combined omics strategies to build a resource of reliably detectable non-canonical translations. In general, our efforts acted at three conceptual levels: tailoring searchable databases towards the needs of non-canonical identification, diversifying sample preparation in order to capture non-canonical translations, and high-end mass spectrometric detection aided by different spectrum matching algorithms to maximize discovery.

At the database level, we relied on concatenation of sequencing-based predictions with the *C. elegans* Ensembl proteome (Figure 1). As such, and in line with similar strategies used in other studies (Chu et al., 2015; Ma et al., 2016b; Budamgunta et al., 2018; Wang et al., 2021), our custom database provides a less biased search space for downstream LC-MS/MS peptide mapping than the proteome database alone, while remaining within reasonable limits of the genome as determined by the transcriptome. However, the percentage of positives is likely to be lower as compared to a well-curated reference proteome such as Ensembl or Uniprot. The issue of theoretical peptide search space size has been addressed before (Borges et al., 2013; NesvizhskiiProteogenomics, 2014; Chatterjee et al., 2016) and with machine learning algorithms these are likely to be circumvented in the near future (Chatterjee et al., 2016; Bouwmeester et al., 2020).

Our results show that a custom database tailored from sequencing-based predictions has a significant impact on novel protein discovery. Using existing *C. elegans* deep proteome datasets in combination with our custom allORF database, we could identify 385 new proteins (Figure 2B) across larval (Xia et al., 2018), adult (Narayan et al., 2016) and stressed (Edifizi et al., 2017) datasets. With extensive MS analysis covering wide physiological stages in *C. elegans*, the number of non-canonical proteins identified was only 0.3% of total predictions, whereas 40% of the Ensembl reference proteome was identified in our meta-analysis. This highlights the challenges in proteome coverage for non-canonical translation products under standard tryptic digest. Furthermore, based on *in silico* digest, 20% of the non-canonical predictions in our allORF database are not expected to produce unique tryptic peptides. Additionally, ribosome sequencing is also prone to detecting regulatory RNA-ribosome interactions that do not correspond to translation of a functional protein, therefore adding spurious predictions to our database (Verbruggen et al., 2021; Ingolia, 2016; Raj et al., 2016). Thus, using existing mass spectrometric tools, standard proteomic sample preparation and a custom database of non-canonical ORF prediction, it is feasible to identify novel proteins, and revisiting available data can be a valuable effort from a discovery perspective.

To test whether the technical pitfalls of standard proteomic workflow could be overcome, we investigated prominent low-MW protein enrichment strategies and alternative digestion against standard tryptic digest (*cf.* Results). Since the median length for predicted sORFs (which comprises approximately 40% of our allORF database) was 25 amino acids (Supplementary Datasheet 5 Figure S1), we expected our own samples relying on endogenous peptidome enrichment strategies (1DRP,2DRP) to yield maximum novel identifications. However, only 24 non-canonical proteins were identified (Figures 4D,E) with a varying length distribution (Supplementary Datasheet 5 Figure S2E) instead of expected <25 amino acids enrichment. Overall, the data from peptidome analysis points towards three possibilities; *1)* sORF-encoded peptides <25 amino acids might be labile, *2)* low abundant and/or *3)* the extraction and enrichment of this group of peptides begs further investigation.

From a discovery perspective, combining different sample preparation methods with enzymatic digestion worked well in our hands. For digested samples, we observed comparatively higher number of PSMs, peptides and proteins identified with tryptic digests (Figure 3A–C, Figure 4C). However, chymotryptic digests identified 42 unique non-canonical proteins that were missed in tryptic digests (Figure 4D). Although more non-canonical proteins are identified in standard digests (WL) as compared to enrichments aimed at targeting 2–12 kDa proteins, a clear advantage is observed with SDS-PAGE and C8-SPE enrichment with unique identifications (Figure 4B), highlighting the complementarity of different sample processing approaches. The non-canonical proteins identified in all sample processing methods span a wide protein length range (Figure 4E, Supplementary Datasheet 5 Figure S2E) although, 96% of the Openprot and sORFs.org predictions are below 100 amino acids (Figure 1). Moreover, we also identified non-canonical proteins of higher MW in the low-MW enriched samples (Figure 4E, Supplementary Datasheet 5 Figure S2E), which could be due to protein instability *in vitro* or *in vivo*. In all, our data supports that alternative approaches for sample processing are complementary to classical tryptic digest in the detection of novel proteins, however, efficient enrichment of endogenous polypeptides below 100 amino acids remains a challenge for the bulk of predicted sORF and altORF annotations. Some studies have used extensive sample fractionation strategies and top-down mass spectrometry (Cardon et al., 2020; Cassidy et al., 2021) to detect non-canonical proteins, however, that is likely to raise the experimental cost and duration by multiple folds. To further chisel the enrichment of low-MW proteins for cost-efficient shotgun proteomics, we generated a strain (LSC 1918) with two HiBit-tag (Schwinn et al., 2020) knock-ins, one at the C-terminus of R06C1.4.1 (84 + 11 amino acids) and one at the predicted C05C9.3 altORF IP_1,500,296 (59 + 11 amino acids). This strain was used for shotgun experiments in this study, and we envision it to be resourceful for further optimization of enrichment for low-MW proteins in the future. Using a blotting system in combination with this strain, sample preparation strategies may be compared for their performance in the low-MW range prior to moving towards LC-MS/MS for unbiased discovery and identification.

Protein identifications are also influenced by the instrumentation and downstream analysis pipeline, and this was the third conceptual level of interest in our current study. Previous work by Shteynberg et al. already highlighted the advantages of combining multiple search algorithms to improve total peptide spectral matches (Shteynberg et al., 2013). Moreover, the recent development of trapped ion mobility coupled with parallel accumulation serial fragmentation on timsTOF platforms (used in this study) have also extended the sensitivity and depth of mass spectrometric data multifold (Meier et al., 2018). However, the downstream analysis pipelines are yet to catch up with the instrumental advances and, to our knowledge, only three analysis pipelines can process the raw files generated by LC-TIMS-MS/MS proteomic experiments (Yu et al., 2020b). We utilized PEAKS and MSFragger to analyse the same data acquired on a timsTOF Pro platform and observed substantial differences in protein identifications (Figure 5). This could be due to various factors, as the underlying algorithms of both search engines differ considerably, however, in combination, that gives an advantage for identification of peptides that might be missed otherwise.

In conclusion, thanks to optimisations at the database, peptide extraction and analysis levels of an omics-based discovery strategy, we can provide mass spectrometric evidence of 467 putative splice variants and 85 novel proteins in *C. elegans*. 18 of these novel proteins were found to be conserved across vertebrates, of which 14 are annotated as ncRNA, 2 mapped onto 5′UTR (uORFs) and 2 are in alternative ORFs in *C. elegans* (Supplementary Datasheet 3, WormBase release WS280). This highlights how genomic annotation can be improved with proteogenomic strategies. These 18 proteins have annotated paralogs in *C. elegans*, indicative of a shared genetic ancestry, however, their functional relevance remains to be investigated. Utilization of sequencing (RNA and Ribosome) in conjunction with proteomics/peptidomics as presented here is likely to further contribute to species-wide genome annotation and our understanding of genetic divergence and compensation. Interestingly, a total of 8 novel proteins belong to uORFs, a regulatory class of proteins (Chew et al., 2016; Johnstone et al., 2016; Zhang et al., 2019) that we propose to define as PEU family (Proteins Encoded in uORFs) for *C. elegans.* Question remains whether living systems tend to follow the robust canonical translation and the non-canonical translations, for large part, are mere slips through reading frames (Verheggen et al., 2017; Chen et al., 2020). However, based on some noteworthy discoveries (Pauli et al., 2014; Anderson et al., 2015; Makarewich et al., 2018; Rathore et al., 2018; Chu et al., 2019; Na et al., 2020), it is certainly clear that nature does not always conform to canonical rules, with discrete prevalence of non-canonical translations, either camouflaged as isoforms/splice variants or anomalous translation of presumed ncRNA, UTRs and polycistronic alternative ORFs. Based on resources like the one presented here, the coming years will certainly witness an increase in functional research into non-canonical translation products and their contribution to organismal phenotypes and plasticity.

## Data Availability

All mass spectrometry raw and spectrum files that are part of this study are available from the MassIVE online repository with identifier MSV000087909.
