# Supplementary material for: Identification of Non-Canonical Translation Products in C. elegans Using Tandem Mass Spectrometry
Source: Front Genet. 2021 Oct 25;12:728900. doi: 10.3389/fgene.2021.728900 (PMC8575065; doi:10.3389/fgene.2021.728900)
Supplement: Supplementary file 3 [file DataSheet5.pdf]

Supplementary material

Identification of non-canonical translation products in *C. elegans* using tandem mass spectrometry.

Bhavesh S. Parmar, Marlies K.R. Peeters, Kurt Boonen, Ellie Cassandra Clark, Geert Baggerman, Gerben Menschaert, Liesbet Temmerman

Contains: supplementary tables 1-4 and supplementary figures 1-3.

LSC1917	R06C1.4.1 crRNA	tgcaatctctttgagtcact
	R06C1.4.1 repair template	cttcaatggacgcaaccttcgtgtcaactacgccaacaaggtgagcggctggcg cctgtttaaaaaaattagctaaactgacaacgccgccgagtgactcaaagagat tgcacttgtgatatacattccaccaaaaattctatcttcgtgc
	R06C1.4.1 fw	cgtcagcaaacactcacgag
	R06C1.4.1 rev	gcggatcccgagatgtaatc
LSC1916	C05C9.3 crRNA	caaggaggagatgtttatgt
	C05C9.3 repair template	aatcgccatcaggtttcctctgttggggatgcttcccacagtgagcggctggcgcct gtttaaaaaaattagctaaacatctcctccttgtagcttgcgcgaattttttttcgg
	C05C9.3 fw	cgacacccctgaatgcttct
	C05C9.3 rev	ccgtaaccttcaagttggcg

Supp Table 1: Oligos used for generating HiBit insertion CRISPR strain used in this study.

**Sequence source**	**Total allORF database**	**small proteome**	**long proteome**	**% small**	**% small of total**	**% long**	**% long of total**	**% total**
Ensembl	25886	1605	24281	1.51	1.16	77.30	17.69	18.86
Ensembl + sORFs.org	2310	710	1600	0.67	0.51	5.09	1.16	1.68
Openprot	54328	50067	4261	47.32	36.49	13.56	3.10	39.59
Openprot + sORFs.org	9670	8404	1266	7.94	6.12	4.03	0.92	7.04
sORFs.org	45000	45000	0	42.53	32.80	0	0	32.80
allORF total	137194	105786	31408		77.10		22.89	100
% total		77.10	22.89					

Supp Table 2: Overview of allORF database composition with number and % of sequence source.

Method abv.	Sample	Cleavage	Enrichment
WL digest	WLT	Trypsin	-
	WLChT	Chymotrypsin	
C8 digest	C8T	Trypsin	C8-reversed phase
	C8ChT	Chymotrypsin	
In-gel digest	GelT	Trypsin	Tris-tricine SDS-PAGE <12kDa
	GelChT	Chymotrypsin	
Undigested	1DRP	Undigested	Acid precipitation, 10,000 Da MW cut-off filtration
	2DRP	Undigested	C8-reversed phase

Supp Table 3: Summary of sample preparation for LC-TIMS-MS/MS in this study. Each sample consists of 4 biological replicates.

	1DRP	2DRP	C8ChT	C8T	GelChT	GelT	WLChT	WLT
MSFragger	324	359	566	1173	775	1512	2090	3318
PEAKS	245	277	446	927	651	1297	1836	2980

Supp Table 4: Total (combined) protein groups identified per sample processing condition with PEAKS and MSFragger

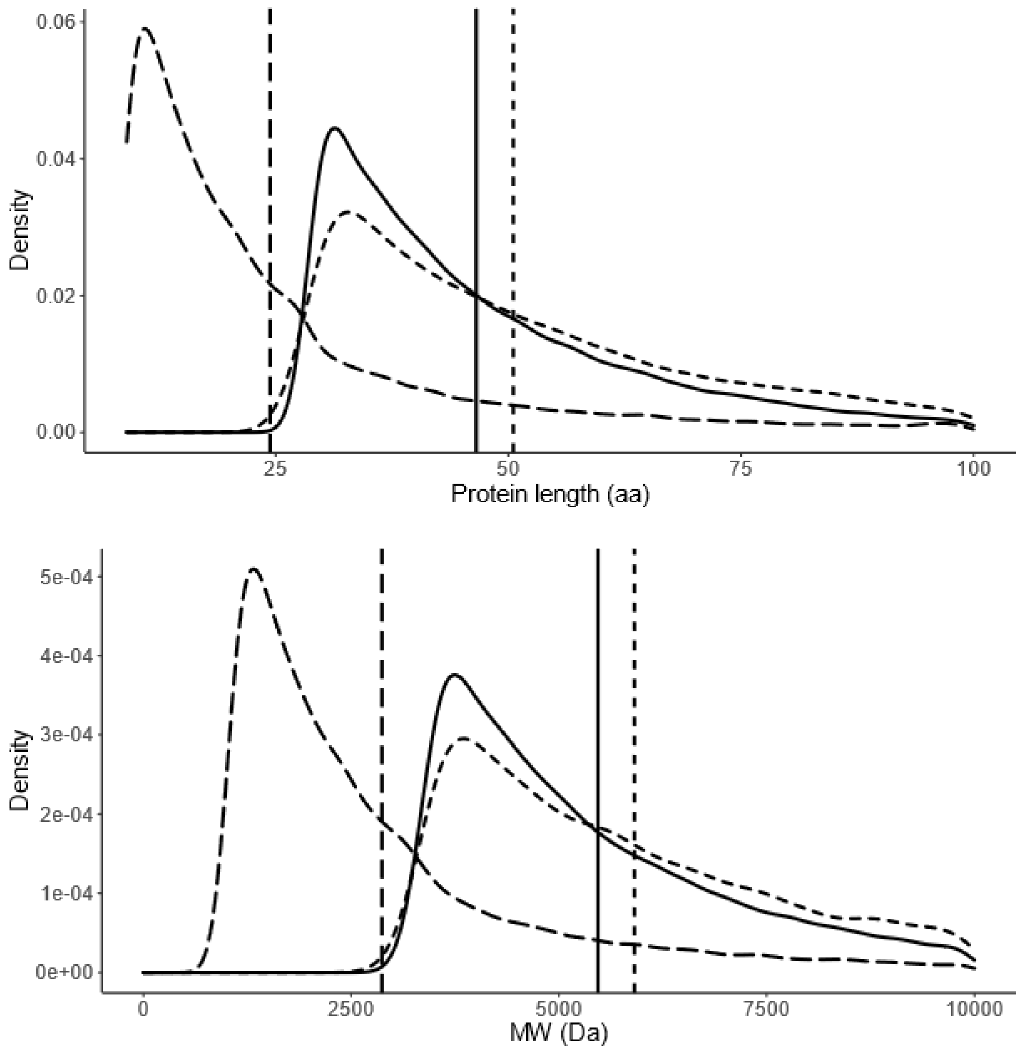

Supp Figure 1: Density plot with median for comparison of sequence length (upper) and molecular weight (lower) for proteins predicted by the sORFs.org pipeline (long dashes), OpenProt pipeline (full line) and by both (short dashes).

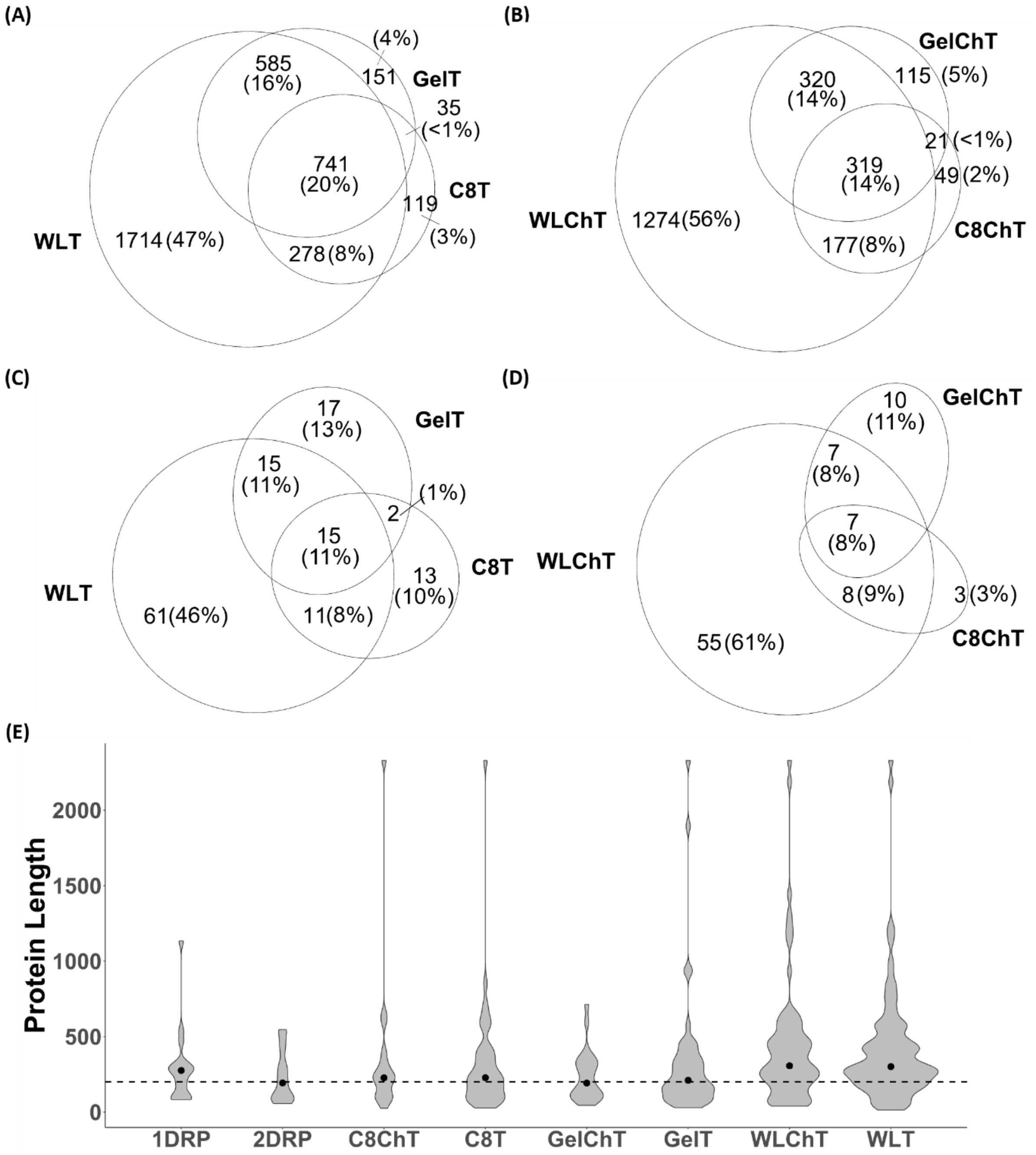

Supp Figure 2: Identification of total proteins (A,B) and non-canonical proteins (C,D p-value:<0.001) influenced by sample processing method; WL: whole lysate, C8: C8-reversed phase enriched, Gel: in-gel digested samples d and enzymatic cleavage; Trypsin (T) (A,C) and Chymotrypsin(ChT) (B,D). (E) Length distribution of non-canonical proteins across all sample processing methods with median dot and dotted line at 200 amino acids($p_{Kruskal\text{-}Wallis}$ 0.01).

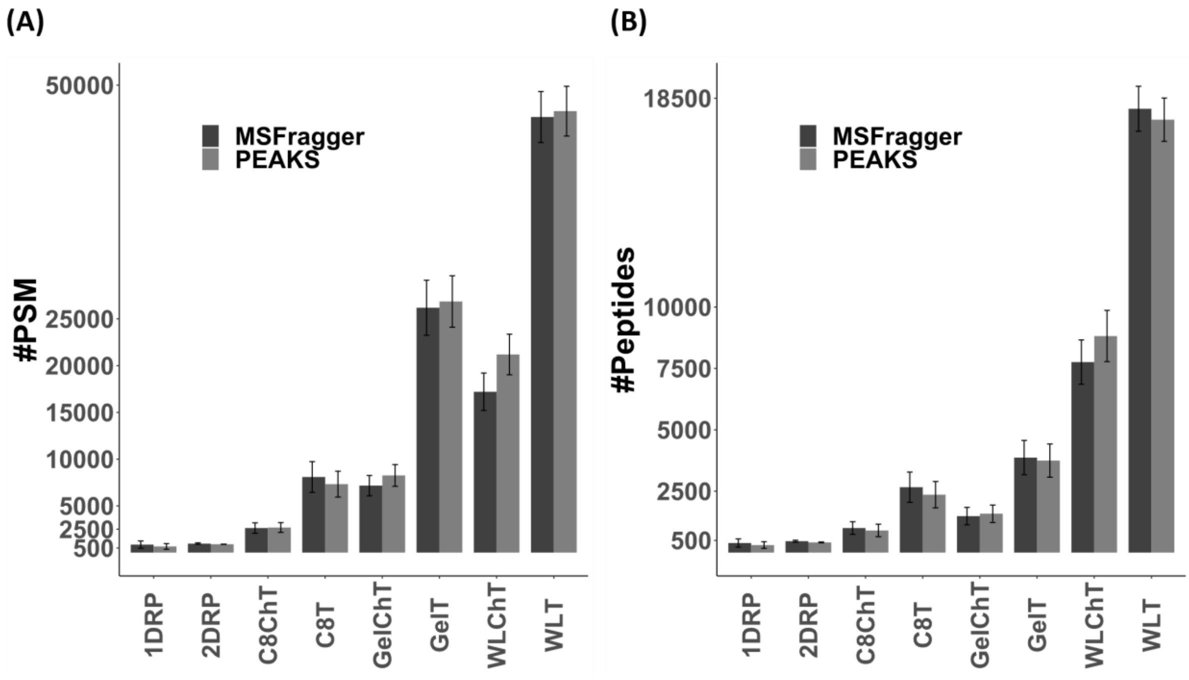

Supp Figure 3: Comparison of mean peptide spectral matches (PSM) (A) and unique peptide sequences (B) identified for each sample processing method used in this study by either MSFragger or PEAKS Online Xpro. Error bars indicate standard deviation between replicates (n=4).
